# *Gandhia* gen. nov.—A New Diatom Genus with Unusual Morphology Split Off from the Genus *Navicula* Bory

**DOI:** 10.3390/plants12233941

**Published:** 2023-11-23

**Authors:** Maxim S. Kulikovskiy, Mital Thacker, Anton M. Glushchenko, Irina V. Kuznetsova, Anton A. Iurmanov, Balasubramanian Karthick, John Patrick Kociolek

**Affiliations:** 1 K.A. Timiryazev Institute of Plant Physiology RAS, IPP RAS, 35 Botanicheskaya St., 127276 Moscow, Russia; glushchenko@ifr.moscow (A.M.G.); pantao@yandex.ru (I.V.K.); yurmanov-anton.ya.ru@yandex.ru (A.A.I.); 2 Biodiversity & Palaeobiology Group, Agharkar Research Institute, Department of Botany, Pune 411004, Savitribhai Phule Pune University, Pune 411007, India; 3 Museum of Natural History, Henderson Building, 15th and Broadway, Department of Ecology and Evolutionary Biology, University of Colorado, Boulder, CO 80309, USA

**Keywords:** diatoms, *Navicula*, *Gandhia*, new genus, morphology, India

## Abstract

A new naviculoid diatom genus, *Gandhia* gen. nov., was described based on a detailed morphological investigation using light and scanning electron microscopy. *Gandhia obtecta* (Jüttner and Cox) Kulikovskiy, Glushchenko, Iurmanov, M.Thacker, B.Karthick and Kociolek comb. nov. was previously described as a member of the genus *Navicula* Bory sensu lato. This species differs from other species in the genus *Navicula* s.l. by the presence of an internal siliceous lamina covering the alveoli and forming the image of longitudinal lines on either side of the axial area, visible in LM. The presence of this siliceous lamina is similar to laminae in genera such as *Pinnularia* and *Gomphoneis*. This unusual morphology is not typical for *Navicula* sensu stricto, as previously noted by other scientists. Additional investigation of *Gandhia obtecta* comb. nov. and *Gandhia ramosissimoides* (H.P. Gandhi) Kulikovskiy, Glushchenko, M.Thacker, B.Karthick and Kociolek comb. nov. from waterbodies of the Western Ghats and the Himalayan region was conducted. Comparison with other species with the same morphological features included two additional species in the genus, namely, *Gandhia jakovljevicii* (Hustedt) Kulikovskiy, Glushchenko, M.Thacker, B.Karthick, and Kociolek comb. nov. and *Gandhia lucida* (Pantocsek) Kulikovskiy, Glushchenko, M.Thacker, B.Karthick and Kociolek comb. nov. We discuss the biogeographic patterns of the species, including disjuncts between Europe and Asia.

## 1. Introduction

The genus *Navicula* Bory was a good example of a catch-all genus, with nearly 10,000 species and subspecific epithets described within it [1]. This genus was one of the largest of all names governed by the Code of Algae, Fungi and Plants. It included many biraphid taxa whose only shared feature was naviculoid symmetry [2]. This genus included species that were later transferred to many new or resurrected genera. Some genera that were separated from *Navicula* sensu lato were shown to be more closely related to taxa within the cymbelloid diatoms rather than the naviculoid diatoms [3,4]. A good example of this is the genus *Placoneis* Mereschkowsky, which has naviculoid symmetry but due to its morphology of pores and cytoplasmic features, has been shown to be more closely related to cymbelloid diatoms. Naviculoid symmetry appears to be a symplesiomorphy, and its utility in diagnosing natural groupings of taxa is limited. Over the last 30 years, many new genera have been described as new and separated from *Navicula* sensu stricto on the basis of such morphological futures as pore occlusions, areola structure, and raphe morphology. These include *Aneumastus* D.G. Mann and Stickle; *Decussata* (Patrick) Lange-Bertalot, *Eolimna* Lange-Bertalot and Schiller; *Fistulifera* Lange-Bertalot; *Mayamaea* Lange-Bertalot; *Prestauroneis* Bruder and Medlin; and many others (see review in Kulikovskiy et al. [2]).

The type species of the genus *Navicula* Bory is *Navicula tripunctata* (O.F. Müller) Bory. This taxon is characterized by naviculoid (boat-shaped) symmetry, a feature shared by all *Navicula* taxa. The raphe of *Navicula* is always straight. Externally, it has hooked ends at the poles and is slightly curved to the secondary side (as demonstrated in the generitype). Internally, the raphe terminates with distinct helictoglossae at the poles and small holes in the center part of the valve. The internal part of the raphe lies on the elevated ridge sometimes referred to as the sternum [5]. The main feature of the genus, as demonstrated by the type species, is areolae in the form of lineolae, that is, areolae characterized by linear narrow openings covered with hymenes internally [2,6,7,8].

Striae are uniseriate in the type species and many others; however, biseriate striae are known in a few species, especially in the *Navicula reinhardtii* (Grunow) Grunow group. Lineolae are longer or of similar length as the interstriae externally and shorter than interstriae internally. Interstriae internally are elevated and form alveoli typical for this genus (according to Lange-Bertalot) [6]. Lange-Bertalot [6], in his circumscription of the genus, noted that alveoli are integrated into an “alveolate system of costae” and “double valve constructions”.

The genus *Navicula* sensu lato was divided into many sections [9,10,11]. *Navicula*, as a genus, according to modern concepts, includes species that were formerly classified in the section “Lineolatae” as defined by P.T. Cleve [12]. This section was raised to a subgenus by Cox [13]. According to the opinion of Round et al. [5], Cox [8,14], and Lange-Bertalot [6], we can describe the genus *Navicula* as naviculoid diatoms with slit-like raphe with ends curved to secondary valve, and with slit-like areolae (lineolae) in uniseriate striae that lie internally within alveoli or easily between broad and elevated interstriae or virgae (sensu Lange-Bertalot) [6]. However, not all species identified as belonging to the “Lineolatae” share just these morphological features. Lange-Bertalot [6] described a new section, Alinea, with species in which central raphe endings are not curved to the secondary side of the valve but to the primary side. The size of this group is rather small in terms of species diversity, but it distinguishes itself from the *Navicula* section by the presence of a centrally curved raphe, as described by Lange-Bertalot [6].

*Hippodonta* Lange-Bertalot, Witkowski, and Metzeltin [15] is a genus sister to the genus *Navicula*, as determined by molecular data [16]. This genus is very similar to species from the section Lineolatae, but *Hippodonta* is characterized by having straight distal raphe ends that do not extend onto the mantle. The raphe is continuous internally and lacks a sternum [2,6]. The genus *Fogedia* Witkowski, Lange-Bertalot, Metzeltin, and Bafana includes species with hyaline areas between striae on the external part of the valve [17,18]. *Fogedia* is a marine genus, but some species are also found in freshwater ecosystems. These species are known from Lake Baikal and belong to the *Navicula lacusbaicali*-group [18]. Species from the *Navicula reinhardtii*-group have biseriate striae that are not typical for *Navicula*. Another unusual species is *Navicula jakovljevicii* Hustedt, which is characterized by the presence of an internal silica lamina covering the alveoli, similar to the condition in the genera *Pinnularia*, *Caloneis*, and *Gomphoneis* [6,19,20].

*Navicula jakovljevicii* was described many decades ago [21]; since, its description has remained in the genus *Navicula* [6]. Morphological investigation of this species showed that this taxon has a silica lamina internally [19]. The occurrence of a silica sheet that covers alveoli internally is atypical in *Navicula*. Subsequently, Gandhi described *Navicula ramosissimoides* with similar morphological features from the Jog Falls, Western Ghats, India [22]. Later, Jüttner et al. [23] described *Navicula obtecta* Jüttner and Cox from Himalayan streams. This species is also characterized by the same morphological feature seen in *N. jakovljevicii*. Jüttner et al. [23] described the morphology of their new species and discussed its taxonomic position. They described their new species and placed it in the genus *Navicula* on the basis of raphe morphology and exterior pore characteristics. However, Jüttner et al. [23] postulated that the interior morphology of their diatom is unusual due to the development of alveolate striae covered by a siliceous lamina extending from the raphe area across the valve towards the margins. This morphological feature is visible in light microscope as longitudinal lines and resembles a similar structure found *Pinnularia* and *Caloneis* [2,6].

The aim of this publication is to provide additional morphological evidence of the naviculoid diatom, *Navicula obtecta*. Furthermore, by utilizing the findings of this study, we propose to establish the novel genus *Gandhia* gen. nov. based on its distinctive morphological features.

## 2. Results and Discussion

**Figure 1:**
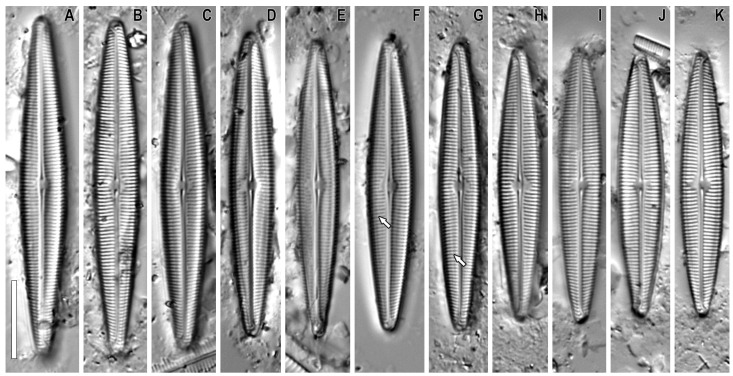
(**A**–**K**). *Gandhia obtecta* (Jüttner and Cox) Kulikovskiy, Glushchenko, Iurmanov, M.Thacker, B.Karthick and Kociolek comb. nov. (**F**,**G**). Valve clearly shows longitudinal lines near the margin on both sides of the axial area (white arrows). Light microscopy, differential interference contrast, size diminution series. Slide no. 33–76 and sample no. AHMA #1639. (**A**–**K**). Valve face, size diminution series. Scale bar = 10 μm. Holotype depicted in (**K**).

**Figure 2:**
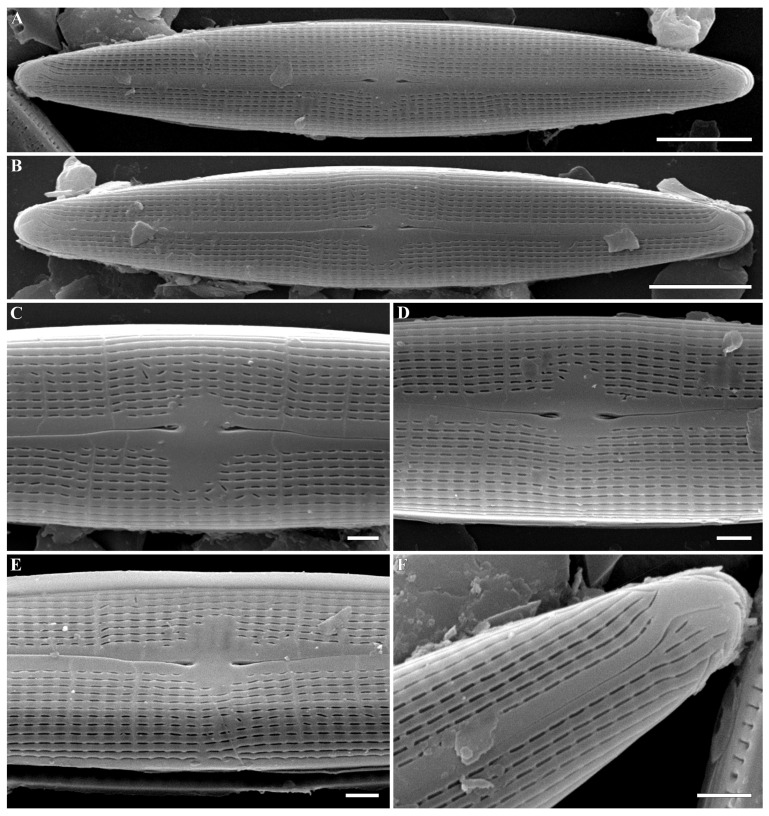
(**A**–**F**). *Gandhia obtecta* (Jüttner and Cox) Kulikovskiy, Glushchenko, Iurmanov, M.Thacker, B.Karthick and Kociolek comb. nov. Scanning electron microscopy, external views. (**A**,**B**). The whole valve. Note the slit-like lineolate striae, dilated central raphe ends, and hooked distal raphe ends. (**C**–**E**). Central area. Dilated central raphe ends are obvious as well as the slit-like striae. (**F**). Valve end. The slit-like areolae appear to be formed by elongated strips of silica. The hooked raphe end is angular and extends to the valve mantle. Scale bar (**A**,**B**) = 5 μm; (**C**–**F**) = 1 μm.

**Figure 3:**
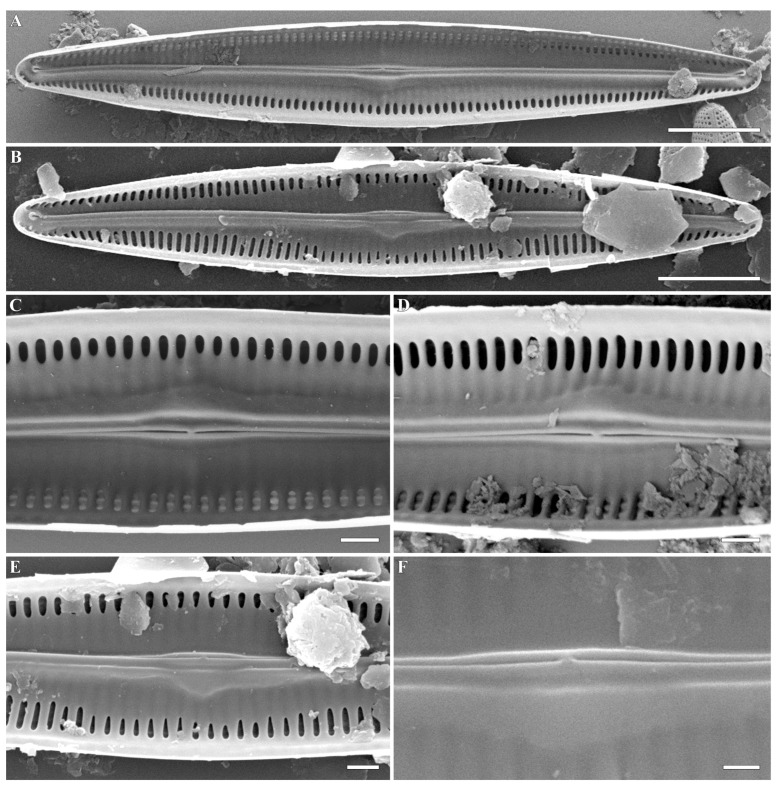
(**A**–**F**). *Gandhia obtecta* (Jüttner and Cox) Kulikovskiy, Glushchenko, Iurmanov, M.Thacker, B.Karthick and Kociolek comb. nov. Scanning electron microscopy, internal views. (**A**,**B**). The whole valve. The siliceous lamina dominates the valve interior, extending from the center towards the margins. (**C**–**F**). Central area. The raphe occurs on an elevated sternum. The siliceous lamina extends over the striae and interstriae. (**C**,**D**). Areolae covered with hymens. The raphe is seen as discontinuous. Scale bar (**A**,**B**) = 5 μm; (**C**–**E**) = 1 μm; (**F**) = 0.5 μm.

**Figure 4:**
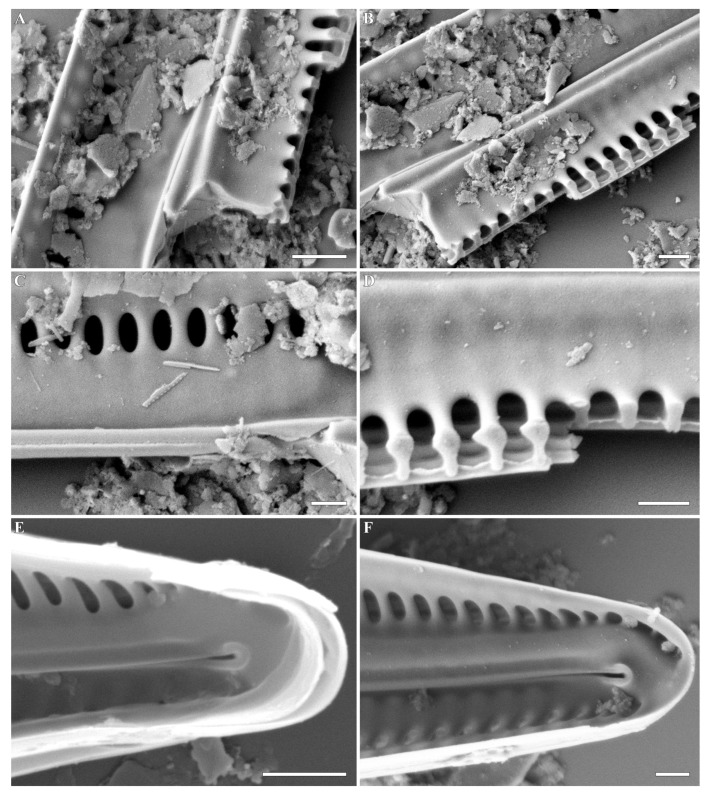
(**A**–**F**). *Gandhia obtecta* (Jüttner and Cox) Kulikovskiy, Glushchenko, Iurmanov, M.Thacker, B.Karthick and Kociolek comb. nov. Scanning electron microscopy, internal views. (**A**–**D**). The thickened nature of the interstriae and the alveoli are evident. (**E**,**F**). Valve ends. The helictoglossae are distinct and have additional thickenings around the raphe terminus towards the valve apex. The alveolate nature of the striae is evident. Scale bar (**A**,**E**) = 1 μm; (**B**–**D**,**F**) = 0.5 μm.

***Gandhia* Kulikovskiy, Glushchenko, Iurmanov, M.Thacker, B.Karthick and Kociolek gen. nov. (Figure 1, Figure 2, Figure 3 and Figure 4)**

Type species (designated here): *Gandhia obtecta* (Jüttner and Cox) Kulikovskiy, Glushchenko, Iurmanov, M.Thacker, B.Karthick and Kociolek comb. nov.

**Description**

**LM** (Figure 1A–K). Valves lanceolate with rounded ends. Length 33.8–42.9 μm, width 5.7–6.3 μm. The axial area is narrow, slightly widening at the center. Raphe filiform is thin and linear. Raphe slits are situated in a narrow axial area. Evident longitudinal lines are present near the margin on both sides of the axial area. The central area is circular, transapically fairly wide. Central endings of raphe are small but dilated and round, curved to the secondary side of the valve. Distal raphe endings are hook-like, extending onto the mantle and turned to the secondary side of the valve. The striae radiate near the center, becoming parallel then becoming slightly convergent towards the apices (21–23 in 10 μm). Lineolae are not observed.

**SEM, external view** (Figure 2A–F). Externally, raphe is straight, slightly undulated near center of the valve. Axial area is narrow, containing a narrow raphe slit. The axial area is not elevated above the striae. The central area is asymmetrical, formed by unevenly shortened striae. Central raphe endings slightly deflected towards the primary side of the valve, with tongue-like insertions. Distal raphe ends forming a hook-like shape, extending on to valve mantle, and turned towards the secondary side. Central raphe endings are dilated but small, comma-like. Striae are parallel to radiate. Areolae in striae near the central area are sometimes not parallel to each other. External openings of the areolae are slit-like, elongated, and sometimes almost confluent, forming longitudinal lines. Interstriae are of the same breadth or less than lineolae.

**SEM, internal view** (Figure 3A–F and Figure 4A–F). Internal axial area is broad. The sternum is narrow and elevated above the striae. Raphe is filiform and running on the elevated sternum. Central raphe endings are straight. Distal raphe endings are slightly deflected and terminate into evident helictoglossae. Central nodule is evident, small, and there is also a smaller, more densely silicified region present on one side. Internally, the striae are alveolate; the proximal two-thirds are covered by a siliceous sheet. Individual areolae are covered internally by prominent hymens.

**Type locality.** Vaddi Falls from Uttara Kannada district of Karnataka state, India. 14.61160° N, 74.55699° E.

**Etymology:** The genus is dedicated to Hemendrakumar Prithivraj Gandhi (20 August 1920–5 June 2008), a well-known diatomist from India.

Habitat: *Gandhia obtecta* was recorded from Vaddi Falls with the following water chemistry measurements: pH, 7.62; electrical conductivity, 87.7 μs cm^−1^; dissolved oxygen, 7.52 ppm; nitrate, 0.01 ppm; phosphate, 3.02 ppm; and water temperature, 23.5 °C.

Distribution: Species of *Gandhia* are distributed across the Peninsular India (Western Ghats and Eastern Ghats), Northeast India, and Nepal. Based on the current distribution range, we expect that the members of this genus will be found across tropical Asia, and further fine-scale studies will offer more insights into the distribution of this genus.

**New combinations:**

***Gandhia obtecta* (Jüttner and Cox) Kulikovskiy, Glushchenko, Iurmanov, M.Thacker, B.Karthick and Kociolek comb. nov.**

Basionym: *Navicula obtecta* Jüttner and Cox in Jüttner, Cox and Ormerod 2000. New or poorly known diatoms from Himalayan streams. Diatom Research. Vol. 15. № 2. P. 246. Figures 15–25. Iconotype here designated is Figure 15.

**Comments:** The specimens in our sample were 37.3–71.0 μm length, 6.5–8.0 μm breadth, and had 21–23 striae in 10 μm and 40–48 lineolae in 10 μm.

***Gandhia jakovljevicii* (Hustedt) Kulikovskiy, Glushchenko, M.Thacker, B.Karthick and Kociolek comb. nov.**

Basionym: *Navicula jakovljevicii* Hustedt 1945. Diatomeen aus Seen und Quellgebieten der Balkan-Halbinsel. Archiv für Hydrobiologie. Suppl. 40. Heft 4. S. 931. Taf. 40. Fig. 17, 18.

***Gandhia lucida* (Pantocsek) Kulikovskiy, Glushchenko, M.Thacker, B.Karthick and Kociolek comb. nov.**

Basionym: *Navicula lucida* Pantocsek 1892, nom. illeg. Beiträge zur Kenntnis der Fossilen Bacillarien Ungarns. Teil III: Süsswasser Bacillarien. Anhang: Analysen 15 neuer Depôts von Bulgarien, Japan, Mähern, Russland und Ungarn. Taf. XVIII. Fig. 264. (non *Navicula lucida* O’Meara 1875).

***Gandhia ramosissimoides* (H.P. Gandhi) Kulikovskiy, Glushchenko, M.Thacker, B.Karthick and Kociolek comb. nov.**

Basionym: *Navicula ramosissimoides* H.P. Gandhi 1970. A further contribution to the diatom flora of the Jog-Falls, Mysore State, India. Beihefte zur Nova Hedwigia. Diatomacee II. Herausgegeben von J. Gerloff und B.J. Cholnoky. Heft 31. S. 777. Fig. 77–79.

*Gandhia obtecta* comb. nov. was described as *Navicula obtecta* from Himalayan streams by Jüttner and Cox in Jüttner et al. [23]. We found this species near the Himalayan region on the southern side of these mountains. In their description of this species, Jüttner and Cox discussed an unusual morphological feature of this taxon, which is a siliceous lamina that covers the alveoli internally on both sides of the central sternum. This is a very interesting and unusual feature for genus *Navicula* sensu stricto as typified by *Navicula tripunctata*. Jüttner and Cox [23] compared their new species with *Gandhia jakovljevicii* comb. nov., previously described as a *Navicula* species by Hustedt [21]. This species shares the same morphological feature of possessing a siliceous lamina covering the alveoli internally. The taxonomic position of the last species was discussed by Hustedt [21], Reichardt [19], Lange-Bertalot [6], and B-Béres et al. [20], who described the morphology of this species using light and scanning electron microscopes. Similarly, another species was documented in Jog Falls, Western Ghats, India, exhibiting the same distinctive morphological characteristics, including the prominent longitudinal lines along the valve margins and the presence of a siliceous lamina covering the alveoli internally. In light of these similarities, we propose a new combination of *Gandhia ramosissimoides* comb. nov., as originally described by Gandhi in 1970.

Lange-Bertalot [6], in his comprehensive monograph, discussed the situation with the taxonomy of species *Navicula* belonging to section Lineolatae. He postulated that the genus *Navicula* sensu stricto with the type species *Navicula tripunctata* is a distinctive genus and differs from other genera. However, some species that were historically described as *Navicula* and, up to now, continue to reside in this genus even in its narrow concept, are really quite different, having morphological features visible via light microscope that differentiates them from other members of the genus. Lange-Bertalot [6] discussed options with regard to the changing generic concept of *Navicula* sensu stricto, suggesting new sections within *Navicula* or describing new genera. Molecular methods have shown that diatoms have evident morphological features that are stable, and these features play important roles in genera delimitation as novelties received during diatom evolution [24,25,26,27,28]. One interesting example of this is the new genus *Karthickia* Kociolek, Glushchenko, and Kulikovskiy which was founded on the basis of morphology [28,29]. *Karthickia verestigmata* Glushchenko, Kulikovskiy, and Kociolek, as a species, resembles *Cymbopleura cuspidata* (Kutzing) Krammer except for the presence of its special stigma morphology. A recent study conducted by our colleagues, involving the isolation of this species in culture and subsequent molecular analysis, has revealed that this particular species is distantly separated from *Cymbopleura* (Krammer) Krammer [30].

Diatoms are unique organisms that form very stable and very complicated silica frustules. These frustules consist of two valves that include many morphological structures such as raphe, areolae, and different pore occlusions, and many additional structures in different phylogenetic groups. Thus, it seems that homoplasy may be common amongst the diatoms for some features [31]. For example, in monoraphid diatoms, structures such as the sinus and cavum [31] are known in unrelated genera [26,31], while homoplastic features in centric diatoms can include rimoportulae, fultoportulae, and others [28,32]. Within naviculoid genera, types of homoplastic features may include stigmata, stigmoids, presence of alveoli, silica laminae, and conopeum, etc. [5]. The foundation for delineating distinct genera lies in the combinations of various morphological characteristics or the presence of unique features. As previously mentioned, numerous morphological structures can emerge across different genera. Similarly, the presence of silica laminae that internally cover the alveoli is a shared trait among the genera *Pinnularia*, *Caloneis*, and *Gomphoneis*, indicating that it is a homoplastic feature within the biraphid diatoms [3,33].

Our investigation of the morphology of *Pinnularia* and *Caloneis* has shown that the silica lamina that covers alveoli is an important feature for the delimitation of different groups between these genera [34,35,36,37]. Our comprehensive investigation of *Pinnularia* and *Caloneis*, utilizing both molecular and morphological data, substantiates the notion that the presence or absence of silica laminae represents a significant distinguishing feature between independent groups within these genera. Additionally, not only the absence or presence of silica laminae but also the breadth of silica laminae and the size of longitudinal lines that are visible in light microscope are important in diagnosing groups within these genera. The same situation is present in the genus *Gomphoneis*, with some morphological groups being distinguished from one another based on the extent of the silica sheets and the position of the corresponding longitudinal lines [33,36]. A taxonomic reevaluation of the genera discussed above will be necessary in the future. Nevertheless, this discussion shows that the presence of silica covering alveoli internally has independently emerged in distinct lineages of pennate diatoms, potentially serving as a valuable tool for differentiation.

*Gandhia obtecta* comb. nov., *Gandhia ramosissimoides* comb. nov., and *Gandhia jakovljevicii* comb. nov. not only exhibit the distinctive feature of possessing silica laminae but also share additional characteristics that distinguish them from *Navicula* sensu stricto. These three species share similar alveoli morphology, with the alveoli being narrow (thus, its number per 10 microns is high). Additionally, in the axial zone, they occasionally approach a nearly confluent arrangement. This feature is similar to another naviculoid genus, *Haslea* Simonsen, which is morphologically similar to the genus *Navicula* but is differentiated from *Navicula tripunctata*. The same structure of pore was observed by us in the very unusual species, *Navicula gogorevii* Chudaev, Glushchenko, Kulikovskiy, and Kociolek from Vietnam [37]. Another important feature that distinguishes the genus *Gandhia* from the genus *Navicula* is the internal structure of the interstriae (virgae). In *Navicula tripunctata* or other typical *Navicula* species (see [2,5,8,18,37]), interstriae are present and slightly elevated on the lineolae roller-like rib. In species of the genus *Gandhia*, interstriae are more complicated, having a stipe and head (resembling a mushroom) that is evident in broken valves (Figure 4B). This interstriae morphology bears a closer resemblance to what is observed in *Pinnularia* or *Caloneis* species, rather than the typical characteristics seen in *Navicula* taxa. Additionally, *Gandhia* species are characterized by having a very broad girdle, a feature lacking in *Navicula* sensu stricto [8,20,23]. Another species that shares all of the morphological features of *Gandhia* is *Navicula lucida* Pantocsek [20]. On the basis of this same morphology, we suggest its transfer to the genus *Gandhiia.* A comparison of four species transferred to the new genus and type species for *Navicula*, *Navicula tripunctata*, is given by us in Table 1. 

*Gandhia lucida* comb. nov. is a fossil diatom known from Miocene sediments in southern Europe [20]. *Gandhia jakovljevicii* comb. nov. is both a fossil and recent taxon from southeast and Central Europe [19,20]. Likewise, *Navicula ramosissimoides* comb. nov. has been described from the Western Ghats [22], and *Gandhia obtecta* comb. nov. is known from recent ecosystems of the Himalayan region. We can hypothesize regarding the spatially disjunct distributions of species within this genus, as well as the evolutionary trajectory of taxa we classify within this genus as an autonomous branch distinct from *Navicula* sensu stricto, dating back to at least the Miocene. It is interesting to note that an axial plate in *Gomphoneis* also originated in the Miocene [3,42].

Disjunct species in southern Europe and Asia were previously shown [30] and discussed by us during a comprehensive investigation of diatoms from Lake Baikal and Southeast Asia. In our discussion of the genus *Navicula*, we explored the occurrence of *Navicula hasta*, a species distinguished by its intriguing morphology. This species was identified in Lake Ohrid, and we also observed a species flock in Lake Baikal exhibiting similar morphological characteristics, such as *Navicula lepskayae* Metzeltin, Kulikovskiy, and Lange-Bertalot [18,43]. Another very interesting example is the widely distributed European species *Navicula reinhardtii* (Grunow) Grunow, known for its distinctive shape and the presence of biseriate striae. This species also shares similarities with the species flock known as *Navicula lacusbaicali* from Lake Baikal, as well as with the species flock of *Navicula reinhardtii* taxa discovered in Mongolia [44].

More pronounced instances of disjunct distributions between Europe and Asia become evident when examining certain taxa at the generic level [30]. In Lake Baikal, we have documented recent species belonging to the genus *Krsticiella* Levkov, which were initially described during the Neogene period in Lake Ohrid [40]. Our investigation showed that species from this genus were known from the Miocene in Lake Baikal [18]. Two monoraphid species from the genus *Trifonovia* Kulikovskiy and Lange-Bertalot are known from recent benthic samples from Lake Baikal and Northern Europe [18]. Equally interesting is the dispersion of *Amphorotia*. Numerous contemporary species have been documented in Lake Baikal, with fossils recorded in China, along with a recent species identified in Vietnam and another in Lake Ladoga, European Russia [45,46,47,48]. Historically, *Amphorotia* was a genus with a broad distribution encompassing Eurasia and North America [45]. Similar distribution patterns have been observed for certain centric diatom taxa, as detailed in [49]. These instances collectively support our hypothesis that numerous diatom groups once had a wide-ranging presence in the past but now exhibit disjunct distributions/disjunct or fragmented geographic ranges. These taxa possess distinctive morphological attributes that have facilitated their classification as new genera, with *Gandhia* gen. nov. serving as an illustrative example of this phenomenon.

## 3. Materials and Methods

**Sampling**. Samples used in this publication were collected by B.Karthick and M.Thacker from the Vaddi Waterfalls (14.61160° N, 74.55699° E) from the Uttara Kannada district of Karnataka state, India.

**Preparation of slides and microscope investigation**. The samples were oxidized by adding an equal volume of concentrated nitric acid (HNO_3_) to eliminate organic matter. Subsequently, the resulting material was alternately centrifuged at 3000 rpm and rinsed with distilled water several times until the sample attained a neutral pH. The cleaned suspension was then air-dried onto coverslips and mounted onto glass slides using Naphrax mounting medium (Refractive Index 1.73). Light microscopy (LM) observations were made at 1000× magnification under oil immersion with an Olympus BX 53 microscope (Tokyo, Japan) equipped with a differential interference contrast optic (Nomarski), and images were captured with an Olympus DP 74 digital camera. For each valve, morphometric measurements were recorded, including length, breadth, and number of striae/10 μm. For scanning electron microscopy observations (SEM), the diatom material was dried onto glass coverslips and affixed to aluminum stubs with double-sided carbon tape. The stubs with the cleaned material were sputter-coated with gold–palladium using an Emitech K57SX sputter coater (Quorum Technologies, Lewes, United Kingdom). SEM observations were performed with an EVO MA15 Carl Zeiss microscope (Oberkochen, Baden-Württemberg, Germany). Samples and slides were deposited in the AHMA, Diatom Section, Pune, India.

## Figures and Tables

**Table 1:** Comparison of species transferred to *Gandhia* gen. nov. and *Navicula tripunctata*.

	*Navicula tripunctata* (O. Müller) Bory 1822	*Gandhia lucida* comb. nov.	*Gandhia jakovljevicii* comb. nov.	*Gandhia obtecta* comb. nov.	*Gandhia obtecta* comb. nov.	*Gandhia ramosissimoides* comb. nov.
References	[2,7]	[7,20,38]	[7,19,21,39,40]	[23,41]	This study	[22]
Distribution	Worldwide. Recent	Romania, Transylvania. Miocene	Southeast and central Europe. Recent	Nepal and India. Recent	India. Recent	India. Recent
Outline	Linear–lanceolate to linear	Lanceolate, gradually tapering to the apex	Lanceolate to elliptic–lanceolate with obtusely to broadly rounded ends	Lanceolate	Lanceolate with rounded ends	Lanceolate with acutely rounded ends
Axial area	Very narrow	Moderately narrow and linear	Narrow, linear	Narrow	Narrow	Narrow
Central area	Almost rectangular, expanded transversely to a little over half the valve width, slightly asymmetric due to the irregular shortening of 2–3 striae on either side	Slightly elliptic	Is small, weakly asymmetrically rounded	Small, more or less circular	Small, circular	Elliptical, fairly wide
Longitudinal lines	Absent	Present	Present	Present	Present	Present
Valve length (μm)	30–70	110–200	32–85	41–60	33.8–42.9	35–48
Valve breadth (μm)	6–10	15–22	7.0–11.7	7.0–9.5	5.7–6.3	4.7–6.3
Striae in 10 μm	9–12	14–16	14–18	21–22	21–23	21–23
Lineolae in 10 μm	32	34–38	29–32	40–45	40–48	No data

## Data Availability

Data are contained within the article.
